# Effects of an *Oroxylum indicum* Extract (Sabroxy^®^) on Cognitive Function in Adults With Self-reported Mild Cognitive Impairment: A Randomized, Double-Blind, Placebo-Controlled Study

**DOI:** 10.3389/fnagi.2021.728360

**Published:** 2021-08-31

**Authors:** Adrian L. Lopresti, Stephen J. Smith, Muhammed Majeed, Peter D. Drummond

**Affiliations:** ^1^Clinical Research Australia, Perth, WA, Australia; ^2^College of Science, Health, Engineering and Education, Murdoch University, Perth, WA, Australia; ^3^Sami-Sabinsa Group Limited, Peenya Industrial Area, Bangalore, India

**Keywords:** *Oroxylum indicum*, mild cognitive impairment, memory, brain-derived neurotrophic factor, randomized clinical trial (RCT)

## Abstract

**Background**: *Oroxylum indicum* has been used in traditional Ayurvedic medicine for the prevention and treatment of several diseases and may have neuroprotective effects.

**Purpose**: Examine the effects of *Oroxylum indicum* on cognitive function in older adults with self-reported cognitive complaints.

**Study Design**: Two-arm, parallel-group, 12-week, randomized, double-blind, placebo-controlled trial.

**Methods**: Eighty-two volunteers received either 500 mg, twice daily of a standardized *Oroxylum indicum extract* or placebo. Outcome measures included several computer-based cognitive tasks, the Control, Autonomy, Self-Realization, and Pleasure scale (CASP-19), Cognitive Failures Questionnaire (CFQ), and the Montreal Cognitive Assessment (MoCA). Changes in the concentration of brain-derived neurotrophic factor (BDNF) were also examined.

**Results**: Compared to the placebo, *Oroxylum indicum* was associated with greater improvements in episodic memory, and on several computer-based cognitive tasks such as immediate word recall and numeric working memory, and a faster rate of learning on the location learning task. However, there were no other significant differences in performance on the other assessed cognitive tests, the MoCA total score, or other self-report questionnaires. BDNF concentrations increased significantly in both groups, with no statistically-significant between-group differences. *Oroxylum indicum* was well tolerated except for an increased tendency for mild digestive complaints and headaches.

**Conclusion**: The results of this first human trial on the cognitive-enhancing effects of *Oroxylum indicum* suggest that it is a promising herbal candidate for the improvement of cognitive function in older adults with self-reported cognitive complaints.

## Introduction

*Oroxylum indicum*, also known as the Indian Trumpet tree, is a small-to-medium size deciduous tree grown in the Asian subcontinent and belonging to the Bignoniacae family. Most parts of the tree have been used in traditional Ayurvedic medicine for the prevention and treatment of several diseases including jaundice, arthritis and rheumatic problems, gastric ulcers, and diabetes (Dinda et al., 2015). In Ayurveda, *Oroxylum indicum* is a component contained in Chyawanprasha and Dashamoolarishta, two multi-herbal formulas commonly used in India (Dev et al., 2010).

Compounds found in the different parts of the *Oroxylum indicum* plant include flavonoids, alkaloids, tannins, glycosides, saponins, phenols, and quinones (Dinda et al., 2015; Nik Salleh et al., 2020). Flavonoids are the major storage components and include baicalein, baicalein-7-O-diglucoside, chrysin, and oroxylin-A (Chen et al., 2003; Yadav et al., 2013). *Oroxylum indicum* and its compounds have several biological activities including anticancer, antibacterial, anti-hyperglycemic, cardioprotective, analgesic, antioxidant, and anti-inflammatory effects (Dinda et al., 2015; Nik Salleh et al., 2020). *Oroxylum indicum* is also reported to possess neuroprotective and anti-epileptic activity (Rathod et al., 2016). In animal models, oroxylin-A, a flavonoid in *Oroxylum indicum*, alleviated hyperactivity, impulsivity, and attentional symptoms associated with attention deficit hyperactivity disorder (Yoon et al., 2008; Dela Pena et al., 2013; Yoon et al., 2013). In a mouse model, an *Oroxylum indicum* extract also prevented cognitive impairment caused by chemotherapy (Pondugula et al., 2020). Moreover, i*n vitro* studies conducted on oroxylin-A have demonstrated that it inhibits dopamine re-uptake (Dela Pena et al., 2013; Yoon et al., 2013), has an antagonistic effect on the gamma-aminobutyric acid (GABA)-A receptor (Kim et al., 2008), and stimulates the expression of brain-derived neurotrophic factor (BDNF; Jeon et al., 2011, 2012). Dopamine and GABA are neurotransmitters believed to play a role in attention, learning, memory, and mood (Kulisevsky, 2000; Mohler, 2009); while BDNF is a protein involved in neuronal survival and growth (Lu et al., 2014). Neuroprotective effects from the flavonoids baicalein (Sowndhararajan et al., 2017; Zhu et al., 2019) and chrysin (Vedagiri and Thangarajan, 2016; Angelopoulou et al., 2020) have also been identified.

Despite its widespread use in Ayurvedic medicine, there are no human trials examining the effects of *Oroxylum indicum* on cognitive function. Due to the promising findings from animal studies and *in vitro* research demonstrating *Oroxylum indicum* can influence several hormones, neurotransmitters, and other biological processes associated with attention, learning, and memory, the aim of this study was to examine its effect on cognitive function in older adults with self-reported cognitive complaints.

## Materials and Methods

### Study Design

**Figure 1:**
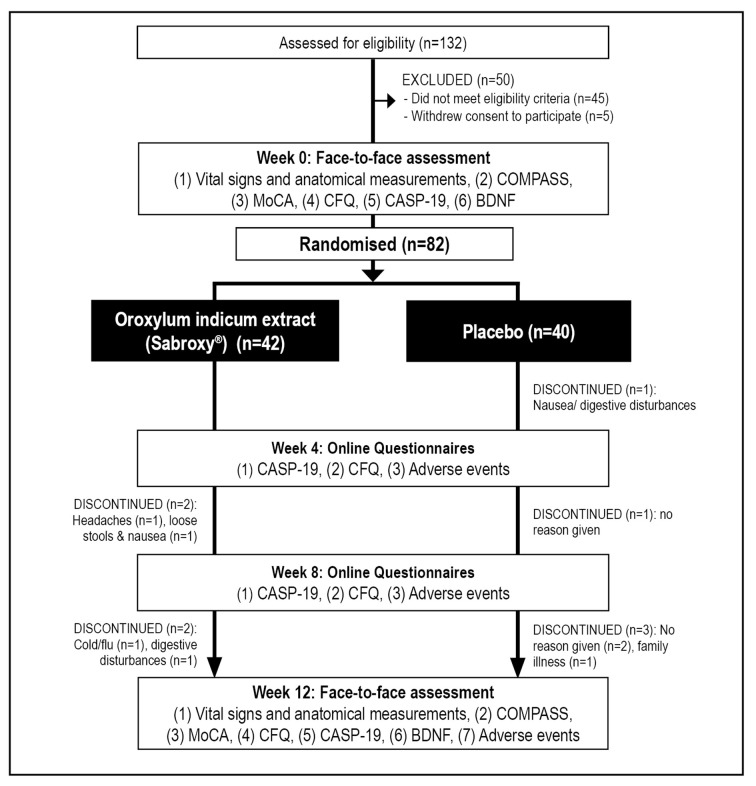
Systematic illustration of study design. BDNF, Brain-derived neurotrophic factor; CASP-19, Control, Autonomy, Self-Realization, and Pleasure; CFQ, Cognitive Failures Questionnaire; COMPASS, Computerized Mental Performance Assessment System; MoCA, Montreal Cognitive Assessment.

This was a two-arm, parallel-group, 12-week, randomized, double-blind, placebo-controlled trial (Figure 1). As there has been no previous human trial on *Oroxylum indicum*, a 12-week treatment period was chosen to assess efficacy, safety, and tolerability in participants. If supplementation is well tolerated and has cognitive-enhancing benefits, it is expected that future trials using a longer treatment duration will be undertaken. The trial protocol was approved by the Human Research Ethics Committee at the National Institute of Integrative Medicine (approval number 0070E_2020) and was prospectively registered with the Australian and New Zealand Clinical Trials Registry (Trial ID. ACTRN12620000800921). As there has been no previous trial examining the effects of *Oroxylum indicum* on cognition, an estimate of effect size could not be determined. However, in previous herbal, cognition trials utilizing the COMPASS as an outcome measure, sample sizes of 70–80 have been used (Kennedy et al., 2019; Wightman et al., 2020). Therefore, we aimed to recruit at least 80 people to determine whether *Oroxylum indicum* produced a similar effect on COMPASS cognitive tasks.

### Recruitment and Randomization

Participants were recruited *via* social media advertisements throughout Western Australia between September and November 2020. Interested volunteers were directed to a website page that provided details about the trial and a link to complete an online screening form for self-reported memory problems; medication use; history of medical or psychiatric disorders; alcohol, nicotine, and other drug use; and supplement and vitamin intake. To assess the severity of depressive symptoms, respondents also completed the Geriatric Depression Scale—Short Form (Van Marwijk et al., 1995). If considered likely eligible, volunteers participated in a telephone interview comprising a structured series of questions to further assess their eligibility and to obtain further demographic details. During this telephone assessment, a researcher administered the Australian adaptation of the Modified Telephone Interview for Cognitive Status (TICS-M; Bentvelzen et al., 2019). The TICS-M is validated against the Mini-Mental State Examination and other cognitive screens and has good sensitivity in detecting mild cognitive impairment (De Jager et al., 2003). Suitable participants were then required to complete an online consent form and attend a face-to-face assessment located at our offices approximately 3–7 days after the phone interview. During the in-person assessment, respondents completed electronic versions of the Control, Autonomy, Self-Realization, and Pleasure questionnaire (CASP-19) and Cognitive Failures Questionnaire (CFQ). A researcher also administered the Montreal Cognitive Assessment (MoCA) and participants completed several tasks from the Computerized Mental Performance Assessment System (COMPASS; see Table 1).

**Table 1:** COMPASS tasks completed.

Task	Description	Scoring
Word presentation	A list of words was displayed on the screen, one word at a time. In this case, 15 words were presented with a display time of 1 s and an inter-stimulus interval of 1 s
Immediate word recall	Participants were given 60 s to write down the words that were presented.	% of words correctly recalled
Picture presentation	A series of 20 photographic images were displayed on the screen, one at a time. The images were presented with a display time of 2 s and an inter-stimulus interval of 1 s.
Computerized location learning	A 5 × 5 grid was presented in which 10 of the squares contained pictures of objects. The participant was asked to remember the locations of these objects within the grid. On five occasions, they were then presented with a blank 5 × 5 grid with the objects displayed to the right of the screen and were required to relocate the objects to the correct location.	Displacement score
Simple reaction time	On 50 occasions, an upwards pointing arrow was displayed on the screen at irregular intervals. Participants responded as quickly as possible when they saw the arrow appear.	Reaction time (ms)
Choice reaction time	Arrows pointing left and right appeared on the screen at irregular intervals. The participant was required to indicate the direction of the arrow as quickly as possible whenever an arrow was displayed, by pressing the corresponding button. In this task, 50 stimuli were presented.	Accuracy (%) and reaction time for the correct responses (ms)
Numeric working memory	A series of numbers was displayed on the screen, one at a time. Participants were required to memorize these numbers. Once the series was complete, numbers were displayed one at a time, and participants were required to indicate if each number was presented in the previous list or not. In this task, three trials were completed with five target numbers in each trial.	Accuracy (%) and reaction time for the correct responses (ms)
Delayed word recall	Participants were instructed to write down the words that were presented to them at the beginning of the assessment. They were given 60 s to complete the task	% of words correctly recalled
Delayed word recognition	All target words that were shown during the word presentation task plus an equal number of decoys were displayed on the screen one at a time. Participants indicated if they remembered seeing the word earlier or not.	Accuracy (%) and reaction time for the correct responses (ms)
Delayed picture recognition	All target pictures shown during the picture presentation task plus an equal number of decoys were displayed on the screen one at a time. Participants indicated if they remembered seeing the picture earlier or not.	Accuracy (%) and reaction time for the correct responses (ms)
Delayed location recognition	A blank 5 × 5 grid was presented with the same objects to the right of the screen as shown in the computerized location learning task. The participant was required to relocate the objects to the position on the grid presented during the computerized locations learning task without seeing them again.	Displacement score

Eligible and consenting participants were randomly allocated to one of two groups (*Oroxylum indicum* extract or placebo). To ensure sequence concealment, a randomization calculator^1^ was used with the randomization structure comprising eight randomly permuted blocks, containing 10 participants per block. The participant identification number was assigned based on the order of participant enrolment in the study. All capsules were packed in identical bottles labeled by two intervention codes (held by the capsule manufacturer until final data collection). Participants and study investigators were blind to treatment group allocation until all outcome data were collected. To compensate participants for their time and travel costs a $30 gift voucher was provided at the end of each face-to-face assessment. Participants allocated to the placebo condition were also offered a free 12-week supply of *Oroxylum indicum* extract capsules.

### Participants

#### Inclusion Criteria

Male and female participants aged 60–85 years, with self-reported impairments in memory or cognitive skills were recruited for this trial as measured by an affirmative response to one of the following questions: (1) Do you feel your memory and thinking has become worse over the past 2–3 years? or; (2) Are you concerned about your decline in memory and thinking? Volunteers had a body mass index (BMI) between 18 and 35 and scored between the 10th and 40th percentile for their age, education, and sex on the Australian adaptation of the Telephone Interview for Cognitive Status-modified version (TICS-M). Participants had no plan to commence new treatments over the study period, were fluent in English and consented (*via* an online consent form) to all pertinent aspects of the trial.

#### Exclusion Criteria

Participants were ineligible to participate in the study if they were diagnosed with dementia (based on the revised National Institute on Aging-Alzheimer’s Association criteria), and suffered from medical conditions including but not limited to: uncontrolled hypertension; myocardial infarction, unstable or severe cardiovascular disease including angina or congestive heart disease in the last year; bleeding disorders; type I diabetes; glaucoma; chronic renal failure; chronic hepatic disease; severe pulmonary disease; gastrointestinal disease requiring regular use of medications; gallbladder disease/gallstones/biliary disease; neurodegenerative or neurological disease. Participants were also ineligible for the study if they were diagnosed with a significant psychiatric disorder, including schizophrenia, bipolar disorder, obsessive-compulsive disorder, personality disorder, or scored greater than 5 on the Geriatric Depression Scale, Short Form (indicating moderate-to-severe depression). A history of stroke, seizures, or head injury (with loss of consciousness), any major surgeries over the last year, significant hearing loss that may impact the person’s ability to complete the phone assessment, regular medication intake including anti-coagulant drugs, anti-cholinergics, acetylcholinesterase inhibitors, and steroid medications, and any change in medication in the last 3 months or an expectation to change during the study period also resulted in exclusion from the study. People taking vitamins or herbal supplements that were reasonably expected to influence study measures, a current or 12-month history of illicit drug abuse, and alcohol intake greater than 14 standard drinks per week were also ineligible to participate in the study.

### Interventions

*Oroxylum indicum* and placebo capsules were identical in appearance, being matched for color coating, shape, and size. The active ingredient, supplied by Sabinsa Corporation, comprised a standardized *Oroxylum indicum extract*, Sabroxy^®^, which is a methanolic extract of the bark of *Oroxylum indicum*. The active treatment contained 500 mg of Sabroxy^®^, which is standardized to contain 10% oroxylin-A, 6% chrysin, and 15% baicalein. The placebo capsules contained the same excipients as the active capsules (cellulose powder, lactose, and magnesium stearate). All participants were instructed to take one capsule, twice daily (morning and evening), with or without food, for 12 weeks. Capsule adherence was assessed by asking participants to provide a capsule count every 4 weeks. Treatment blinding was assessed by asking participants to predict group allocation (placebo, *Oroxylum indicum*, or unsure) at the end of the study. All capsules were provided to participants with directions for use provided on capsule bottles. An information sheet about capsule intake and what to do if a dose was missed was also given to participants. This information was also conveyed to participants during their initial in-person assessment.

### Outcome Measures

#### Primary Outcome Measure

##### Computerized Mental Performance Assessment System (COMPASS)

The COMPASS (Northumbria University, Newcastle upon Tyne, UK) is a software application for the presentation of computerized standard and novel cognitive tasks that assess episodic memory and speed of response. Results on the COMPASS are sensitive to dietary interventions and nutritional supplementation (Cox et al., 2014; Dodd et al., 2015; Kennedy et al., 2017). The cognitive tasks used in this study are detailed in Table 1. The COMPASS was completed at baseline and week 12. At each assessment, participants completed a brief practice session to familiarize themselves with the necessary tasks and then completed the full COMPASS assessment as detailed in Table 1. Since the goal of this study was to examine the chronic effects of supplementation, participants were asked to not take their morning study medication on the day of his/her final assessment. Moreover, they arrived for testing in a fasting state and did not consume any morning caffeinated beverage. Participants were also asked to avoid consuming any alcohol on the evening before testing. All assessments occurred in the morning between 8 and 11 am, with the time of testing at baseline and week 12 occurring at approximately the same time for each participant. When participants arrived for testing, a blood sample was collected, they consumed a breakfast bar and non-caffeinated herbal tea (optional) while they completed their online questionnaires, and then completed the COMPASS testing.

#### Secondary Outcome Measures

##### Control, Autonomy, Self-realization, and Pleasure (CASP-19)

The CASP-19 is a measure of well-being developed for older people, spanning four domains of control, autonomy, self-realization, and pleasure (Hyde et al., 2003). Items on this 19-item questionnaire are rated on a 4-point Likert scale ranging from never to often, with higher scores indicating better well-being. In studies on community-dwelling older-age adults, and older adults with dementia, the CASP-19 demonstrated sound psychometric properties (Sim et al., 2011; Stoner et al., 2019). The CASP-19 was completed at baseline, and weeks 4, 8, and 12.

##### Cognitive Failures Questionnaire (CFQ)

The 25-item CFQ assesses the frequency with which people experienced cognitive failures, such as absent-mindedness in everyday life, slips, and errors of perception, memory, and motor functioning (Broadbent et al., 1982). The CFQ has good psychometric properties (Bridger et al., 2013) where higher scores indicate worsened cognitive skills. The CFQ was completed at baseline, and weeks 4, 8, and 12.

##### Montreal Cognitive Assessment (MoCA)

The MoCA is a validated measure of mild cognitive impairment assessing short–term memory, visuospatial abilities, executive function, attention, concentration, working memory, language, and orientation to time and place (Nasreddine et al., 2005). The MoCA has good psychometric properties and is regularly used as a screening tool for mild cognitive impairment and Alzheimer’s disease (Abd Razak et al., 2019; Bruijnen et al., 2020). In addition to a total score, a memory index score (a measure of delayed word recall) can also be calculated which has been shown to be predictive of short-term conversion to Alzheimer’s disease (Julayanont et al., 2014). The MoCA was completed at baseline and week 12.

##### Brain-Derived Neurotrophic Factor (BDNF)

BDNF is a key molecule involved in plastic changes related to learning and memory. Changes in BDNF expression are associated with both normal and pathological aging, psychiatric diseases, and the regulation of plastic changes in the adult brain (Miranda et al., 2019). Blood samples for BDNF analysis were collected at baseline and week 12.

Blood samples were collected in EDTA tubes after an overnight fast. All samples were collected between 9 and 11 am. EDTA tubes were centrifuged at 1,500× *g* for 15 min and the plasma was stored in a −80-degree freezer until later analysis. On the day of assay, serum samples were thawed and mature BDNF measured using the commercially available RayBio human BDNF enzyme-linked immunosorbent assay (ELISA) kit (RayBiotech Life, Peachtree Corners, GA, USA) according to the manufacturer instructions. Serum samples were diluted 1:250 prior to assay. The BDNF ELISA is an indirect sandwich kit in which BDNF from the test sample is captured by an immobilized BDNF antibody and after several washes, a biotinylated anti-human BDNF antibody is added. After washing away unbound biotinylated antibody horseradish peroxidase conjugated streptavidin was added. The wells were again washed before the addition of TMB substrate. The amount of BDNF in the test sample is directly proportional to developed color measured by optical density.

##### Adverse Events

Tolerability and safety of capsule intake by participants were assessed at weeks 4, 8, and 12 *via* an online question querying adverse effects that were believed to be associated with capsule intake. Participants were also requested to contact the researchers immediately if any adverse effects were experienced.

### Statistical Analysis

For baseline data, an independent samples *t*-test was used to compare group data for continuous variables, and a Pearson’s Chi-square test was used to compare categorical data. Three separate general linear model multivariate ANOVAs were conducted to assess the change in scores on the self-report questionnaires (CASP-19 sub-scale scores and CFQ total score, weeks 0, 4, 8, and 12); and episodic memory and speed of performance (baseline and week-12 scores). Medication and nutraceutical use were included as covariates for the analysis of episodic memory and speed of performance. Episodic memory comprised scores on the following measures: immediate word recall (percentage correct), delayed word recall (percentage correct), location learning recall (displacement score), word recognition (percentage correct), picture recognition (percentage correct), numeric working memory (percentage correct), and MoCA memory index score. To maintain consistency in scoring (i.e., increased scores indicate improvements in cognitive performance), displacement scores on the location learning recall task were converted from positive to negative numbers with a maximum score of 0. Speed of performance comprised the following measures: simple reaction time, choice reaction time, numeric working memory reaction time, and picture recognition reaction time (reaction time in ms of correct responses). These cognitive skill categorizations are consistent with other studies that have used the COMPASS as an outcome measure (Kennedy et al., 2019; Wightman et al., 2020). Univariate ANOVAs were also conducted to examine between-group differences in changes in the MoCA total score, MoCA memory index score, and BDNF concentrations (medication and nutraceutical use were entered as covariates). To avoid problems of collinearity, a multivariate analysis was not conducted on the two MoCA scores. As a measure of visuospatial learning, a time × trial × group, repeated-measures ANOVA was conducted on the displacement scores (trials 1–5) on the location learning task. A Cohen’s d effect size was also calculated for all individual outcome measures. The normality of residuals was assessed by the visual inspection of Q-Q plots and analysis of skewness and kurtosis. This demonstrated that most self-report data and BDNF concentrations were normally distributed. However, as COMPASS scores were not normally distributed, the data were winsorized whereby scores greater than three standard deviations from the mean were replaced with the next highest score. Winsorizing is a robust method to normalize data (Cleophas, 2015) and improved the normality of COMPASS data. Where necessary, degrees of freedom were adjusted using the Greenhouse-Geisser approach to correct for violations of the sphericity assumption. Data from participants were included in the analyses of self-report outcomes if questionnaire data were obtained at week 4 (last observation carried forward from week 4 for missing values). All data were analyzed using SPSS (version 26; IBM, Armonk, NY, USA). The critical *p*-value was set at *p* ≤ 0.05 for all analyses. Due to the exploratory nature of this study, a correction to the p-value due to multiple testing was not undertaken. However, the type 1 error rate was minimized by using a step-down analysis, whereby the multivariate ANOVA needed to be significant before proceeding to the exploration of univariate analyses.

## Results

### Study Population

#### Baseline Questionnaire and Demographic Information

As detailed in Figure 1, from 132 people who completed the initial online screening questionnaire, 45 individuals did not meet the eligibility criteria, and five individuals withdrew consent to participate in the study. 82 volunteers participated in the study and 73 people completed the study. Pre and post blood samples were collected from 70 participants. Details of participant demographic information and baseline scores of the total recruited sample are detailed in Tables s 2, 3. Baseline demographic details, questionnaire responses, and COMPASS test results were equivalent in both groups. Nine participants withdrew from the study. Reasons for withdrawal included no reason given (*n* = 3), nausea/digestive complaints (*n* = 3), headaches (*n* = 1), cold/flu (*n* = 1), and illness of a family member (*n* = 1).

**Table 2:** Baseline demographics details and questionnaire scores.

		Placebo (*n* = 40)	*Oroxylum indicum* (*n* = 42)	*p*-value
Age	Mean	68.28	66.07	0.061^a^
	SE	0.9	0.73
Gender	Female (*n*)	28	33	0.374^b^
	Male (*n*)	12	9
BMI	Mean	25.88	26.38	0.541^a^
	SE	0.59	0.56
Systolic blood pressure (mmHg)	Mean	129.08	129.24	0.965^a^
	SE	2.75	2.12
Diastolic blood pressure (mmHg)	Mean	79.41	81.00	0.505^a^
	SE	1.84	1.51
Marital status	Single	9	16	0.125^b^
	Married/de facto	31	26
Educational level	Secondary	21	22	0.733^b^
	Tertiary	11	14
	Post-graduate	8	6
Exercise level (*n*)	Never/rarely	5	7	
	1–2 times a week	8	3
	3–5 times a week	15	12	0.180^b^
	6+ times a week	12	20
Taking any prescription medication	Yes	19 (47.50%)	21 (50.00%)	0.821^b^
Taking statin	Yes	9 (22.50%)	7 (16.67%)	0.951^b^
Taking antidepressant	Yes	6 (15.00%)	3 (7.14%)	0.255^b^
Taking proton pump inhibitor	Yes	5 (12.50%)	3 (7.14%)	0.414^b^
Taking nutraceutical/ phytoceutical	Yes	13 (32.50%)	11 (26.19%)	0.505^b^
MoCA total score	Mean	25.18	24.60	0.301^a^
	SE	0.41	0.37
MoCA Memory Index Score	Mean	9.98	8.76	0.096^a^
	SE	0.53	0.49
CFQ—Total score	Mean	42.23	42.81	0.798^a^
	SE	1.53	1.68
CASP-19—Control	Mean	8.15	7.31	0.056^a^
	SE	0.32	0.29
CASP-19—Autonomy	Mean	10.68	10.52	0.748^a^
	SE	0.38	0.40
CASP-19—Pleasure	Mean	13.73	13.38	0.379^a^
	SE	0.28	0.27
CASP-19—Self realization	Mean	11.65	11.19	0.362^a^
	SE	0.37	0.34
BDNF (ng/ml)	Mean	952.00	863.33	0.104^a^
	SE	41.32	33.61

*a, independent samples *t*-test; b, chi-square analysis*.

**Table 3:** Baseline COMPASS scores.

		Placebo (*n* = 40)	*Oroxylum indicum* (*n* = 42)	*p*-value^a^
Immediate word recall (%)	Mean	30.17	30.79	0.807
	SE	1.85	1.77
Delayed word recall (%)	Mean	15.00	16.98	0.483
	SE	0.59	0.56
Systolic blood pressure (mmHg)	Mean	129.08	129.24	0.965^a^
	SE	2.04	1.94
Simple reaction time (ms)	Mean	383.39	395.31	0.562
	SE	15.05	13.93
Choice reaction time correct (%)	Mean	97.50	98.24	0.223
	SE	0.46	0.39
Choice reaction time for correct responses (ms)	Mean	598.33	617.71	0.393
	SE	17.03	14.87
Location learning trial 1 (displacement score)	Mean	17.58	18.17	0.686
	SE	1.07	0.99
Location learning trial 2 (displacement score)	Mean	13.93	13.29	0.703
	SE	1.10	1.25
Location learning trial 3 (displacement score)	Mean	9.73	8.93	0.641
	SE	1.24	1.17
Location learning trial 4 (displacement score)	Mean	7.28	6.52	0.637
	SE	1.08	1.15
Location learning trial 5 (displacement score)	Mean	5.70	3.67	0.090
	SE	0.86	0.82
Location learning recall (displacement score)	Mean	7.10	5.81	0.387
	SE	1.02	1.08
Numeric working memory correct (%)	Mean	91.92	89.23	0.158
	SE	1.17	1.46
Numeric working memory reaction time for correct responses (ms)	Mean	1,213.38	1,355.70	0.069
	SE	54.38	54.85
Word recognition correct (%)	Mean	75.39	73.17	0.321
	SE	1.50	1.61
Word recognition reaction time for correct responses (ms)	Mean	1,384.76	1,572.62	0.133
	SE	80.66	92.79
Picture recognition correct (%)	Mean	93.19	95.67	0.057
	SE	1.11	0.64
Picture recognition reaction time for correct responses (ms)	Mean	1,111.88	1,130.42	0.757
	SE	47.88	35.92

*a, independent samples *t*-test*.

### Outcome Measures

#### Primary Outcome Measure: COMPASS Scores

Changes in the COMPASS tasks and cognitive categories across the two treatment groups and ANOVA significance levels are detailed in Table 4. A general linear model multivariate ANOVA revealed there was a statistically significant between-group difference for episodic memory (*F*_(7,59)_ = 2.77, *p* = 0.015) but not speed of performance (*F*_(5,61)_ = 1.03, *p* = 0.410). Episodic memory [comprising the scores from immediate word recall (percentage correct), delayed word recall (percentage correct), location learning recall (displacement score), word recognition (percentage correct), picture recognition (percentage correct), numeric working memory (percentage correct), and MoCA memory index score] changed in the *Oroxylum indicum* (*F*_(7,28)_ = 2.38, *p* = 0.048) and placebo groups (*F*_(7,27)_ = 2.94, *p* = 0.020); however, increases were greater in the *Oroxylum indicum* group.

**Figure 2:**
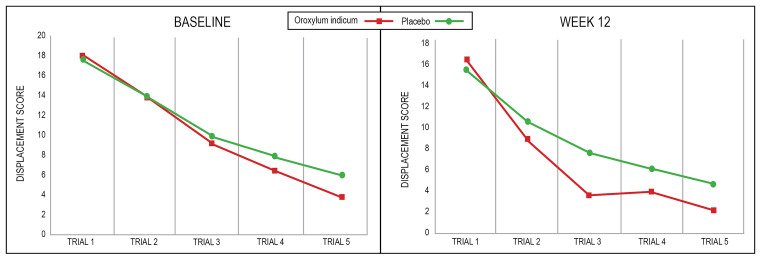
Displacement scores in the COMPASS location learning task.

An examination of individual COMPASS tasks revealed greater increases in the correct responses in immediate word recall (*F*_(1,65)_ = 5.94, *p* = 0.018) and numeric working memory (*F*_(1,65)_ = 4.08, *p* = 0.048) in the *Oroxylum indicum* group compared to the placebo group. There were no other statistically significant between-group interactions on other COMPASS tasks detailed in Table 4. In the location learning task (comprising five learning trials), a repeated-measures ANOVA time × group × trial analysis revealed a non-significant interaction (*F*_(4,210)_ = 1.21, *p* = 0.306; Table 5). However, a statistically significant time × trial × group quadratic component was identified indicating a faster rate of learning in the *Oroxylum indicum* group compared to the placebo group at week 12 and both groups at baseline (*F*_(1,71)_ = 4.16, *p* = 0.045; Figure 2).

**Table 4:** Change in COMPASS tasks.

		Placebo (*n* = 35)				*Oroxylum indicum* (*n* = 38)				Cohen’s d effect size	Univariate between-group *p*-value^b^	Multivariate between-group *p*-value^b^
		Week 0	Week 12	Change	*p*-value^a^	Week 0	Week 12	Change	*p*-value^a^			
**Measures of episodic memory**												
Immediate word recall (%)	Mean	31.05	30.10	−0.95	0.548	31.58	37.02	5.44	0.029	0.52	0.018	0.015
	SE	2.03	2.23	1.90	1.91	1.94	2.16
Delayed word recall (%)	Mean	16.19	16.19	0.00	1.00	17.90	22.28	4.39	0.065	0.40	0.116
	SE	2.23	2.10	1.62	2.08	1.78	1.97
Location learning recall (displacement score)	Mean	−7.54	−5.09	2.46	0.011	−5.87	−2.89	2.97	0.011	0.09	0.917
	SE	1.13	0.89	0.93	1.09	0.70	1.00
Word recognition correct (%)	Mean	75.69	75.98	0.29	0.888	72.97	77.39	4.41	0.136	0.30	0.273
	SE	1.67	2.19	2.07	1.63	1.90	2.52
Picture recognition correct (%)	Mean	92.64	95.57	2.93	0.010	95.57	96.50	0.93	0.226	0.39	0.182
	SE	1.23	0.75	0.99	0.72	0.63	0.75
Numeric working memory correct (%)	Mean	92.92	92.45	0.48	0.466	89.24	92.57	3.33	0.071	0.44	0.048
	SE	1.11	1.03	0.93	1.60	1.07	1.46
MoCA—Memory index score	Mean	10.29	10.09	−0.20	0.765	8.87	9.97	1.11	0.017	0.42	0.081
	SE	0.54	0.51	0.55	0.50	0.54	0.46
**Measures of speed of performance**												
Simple reaction time (ms)	Mean	376.05	359.23	−16.82	0.220	388.74	365.77	−22.97	0.080	0.08	0.851	0.410
	SE	15.48	9.60	13.43	14.26	10.28	11.90
Choice reaction time for correct responses (ms)	Mean	597.42	558.28	−39.15	0.014	604.73	573.83	−30.90	0.018	0.10	0.969
	SE	19.41	13.74	13.58	14.41	11.38	12.63
Numeric working memory reaction time for correct responses (ms)	Mean	1,185.61	1,134.66	−50.95	0.145	1,347.41	1,190.91	−156.50	0.006	0.44	0.110
	SE	56.15	52.01	31.20	60.30	44.15	45.86
Word recognition reaction time for correct responses (ms)	Mean	1,346.87	1,275.78	−71.09	0.297	1,542.72	1,389.05	−153.67	0.061	0.21	0.504
	SE	85.54	73.28	67.11	100.60	87.01	66.75
Picture recognition reaction time for correct responses (ms)	Mean	1,084.06	1,063.97	−20.08	0.878	1,101.60	1,055.86	−45.74	0.059	0.15	0.091
	SE	48.40	40.07	33.93	35.18	36.63	2.44
**Other COMPASS tasks**												
Choice reaction time correct (%)	Mean	97.37	97.54	0.17	0.707	98.11	98.00	−0.11	0.803	0.11	0.603	NA
	SE	0.50	0.42	0.45	0.43	0.33	0.43

^a^Repeated measures ANOVA; ^b^ANOVA with medication and supplement use entered as covariates.

**Table 5:** Change in COMPASS location learning scores.

		Placebo (*n* = 35)		*Oroxylum indicum* (*n* = 38)		Time × trial × group interaction	Time × trial × group interaction (linear)	Time × trial × group interaction (quadratic)
Location learning trial 1 (displacement score)	Mean	17.57	15.71	18.16	16.63			
	SE	1.18	1.13	1.08	1.44			
Location learning trial 2 (displacement score)	Mean	13.86	10.63	14.00	8.97			
	SE	1.17	1.16	1.31	1.03			
Location learning trial 3 (displacement score)	Mean	9.97	7.66	9.29	3.66	0.306	0.993	0.045
	SE	1.36	1.08	1.22	0.68			
Location learning trial 4 (displacement score)	Mean	7.86	6.06	6.45	4.05			
	SE	1.19	1.12	1.13	0.88			
Location learning trial 5 (displacement score)	Mean	5.97	4.71	3.74	2.18			
	SE	0.95	1.06	0.86	0.71			

#### Secondary Outcome Measures: CFQ and CASP-19 Sub-scale Scores

Changes in the self-report questionnaire scores across the two treatment groups and repeated measures ANOVA significance levels are detailed in Table 6. A multivariate analysis revealed there was a statistically-significant time effect (*F*_5,15_ = 1.95, *p* = 0.034) but no statistically-significant time × group (*F*_5,15_ = 1.07, *p* = 0.402), or main effect for group (*F*_5,15_ = 0.12, *p* = 0.987). An examination of individual scores revealed a significant time × group effect for the CASP-19 control score only (*F*_3,209_ = 3.45, *p* = 0.022). CASP-19 control scores increased significantly in the *Oroxylum indicum* (*F*_3,123_ = 2.70, *p* = 0.048) and there was a non-significant increase in the placebo group (*F*_2.5,95_ = 2.64, *p* = 0.064).

**Table 6:** Change in questionnaire scores and BDNF concentrations.

		Placebo (*n* = 39)					*Oroxylum indicum* (*n* = 42)					*p*-value	Cohen’s d effect size^c^	Multivariate analysis^d^		
		Week 0	Week 4	Week 8	Week 12	*p*-value^a^	Week 0	Week 4	Week 8	Week 12	*p*-value^a^			Group Main effects	Time effects	Time × group interaction
CASP-19—Control	Mean	8.13	8.31	7.64	7.79	0.064	7.31	7.93	7.88	7.90	0.048	0.022^b^	0.50	0.987	0.034	0.402
	SE	0.33	0.32	0.32	0.32	0.29	0.35	0.31	0.35
CASP-19—Autonomy	Mean	10.69	10.87	11.08	10.90	0.669	10.52	11.00	10.98	11.21	0.179	0.645^b^	0.21
	SE	0.39	0.35	0.36	0.37	0.40	0.38	0.38	0.32
CASP-19—Pleasure	Mean	13.69	13.64	13.56	13.67	0.871	13.38	13.64	13.33	13.69	0.230	0.560^b^	0.24
	SE	0.28	0.29	0.30	0.32	0.27	0.27	0.30	0.24
CASP-19—Self Realization	Mean	11.56	11.56	11.56	11.82	0.600	11.19	11.48	11.40	11.76	0.170	0.806^b^	0.19
	SE	0.37	0.38	0.37	0.39	0.34	0.39	0.33	0.33
CFQ—Total	Mean	42.49	40.34	39.97	38.43	0.031	42.95	38.71	39.13	38.05	0.022	0.740^b^	0.09
	SE	1.69	1.92	1.61	1.72	1.83	1.84	1.84	1.81
		Placebo (*n* = 35)					*Oroxylum indicum (*n* = 38)*				

MoCA– total score	Mean	25.29	−	−	25.40	0.768^e	24.79	−	−	25.24	0.238^e^	0.525^f^	0.15	−	−	−
	SE	0.45	−	−	0.46	0.39	−	−	0.41
		Placebo (*n* = 34)					*Oroxylum indicum (*n* = 36)*				

BDNF (ng/ml)	Mean	863.33	−	−	1058.80	0.001^e	953.02	−	−	1211.58		#x0003C;0.001^e^	0.441^f^	0.17	−	−	−
	SE	33.61	−	−	57.78	42.47	−	−	54.63

*^a^Repeated measures ANOVA within-group, time effects; ^b^repeated-measures ANOVA, univariate time × group interaction; ^c^Placebo vs. Oroxylum indicum Cohen’s d effect size (based on changes in scores from baseline to week 12); ^d^repeated-measures ANOVA multivariate *p*-value; ^e^paired-samples *t*-test; ^f^general linear model univariate ANOVA with medication and supplement use entered as covariates*.

#### Secondary Outcome Measure: MoCA

Changes in the MoCA scores across the two treatment groups and repeated-measures ANOVA significance levels are detailed in Tables s 4 and 6. A repeated-measures ANOVA demonstrated that there was no statistically significant change over time on the MoCA total score (*F*_1,71_ = 1.01, *p* = 0.536) or the MoCA memory index score (*F*_1,71_ = 3.23, *p* = 0.077).

#### Secondary Outcome Measure: BDNF Concentrations

Changes in the BDNF concentrations across the two treatment groups and ANOVA significance levels are detailed in Table 6. An ANOVA demonstrated that there was no statistically significant between-group effect (*F*_1,66_ = 0.601, *p* = 0.441). In both the *Oroxylum indicum* (*F*_1,35_ = 21.20, *p* < 0.001), and placebo (*F*_1,33_ = 13.42, *p* = 0.001) groups, BDNF concentrations increased over time. An exploratory analysis revealed that for all participants (placebo and *Oroxylum indicum*) increases in BDNF were not significantly correlated with improvements in any outcome measure.

#### Intake of Supplements

At weeks 4 and 8, participants reported the number of remaining capsules. Capsule bottles with remaining capsules were also returned on the week 12 assessment. Based on these details, 93% of participants took more than 80% of their capsules.

#### Efficacy of Participant Blinding

To evaluate the efficacy of condition concealment throughout the study, participants were asked at the end of the study to predict condition allocation (i.e., placebo, *Oroxylum indicum*, or unsure). The efficacy of group concealment was high as 79% of participants either incorrectly guessed treatment allocation or were unsure.

#### Adverse Events

The frequency of self-reported adverse events is detailed in Table 7. There was a tendency for more reported adverse events in the *Oroxylum indicum* group (*n* = 10) compared to the placebo group (*n* = 6). No serious adverse events were reported by participants, although one participant in the *Oroxylum indicum* group withdrew due to reported ongoing headaches. In the *Oroxylum indicum* group, the majority of adverse events were digestion-related complaints (e.g., loose stools, nausea, and bloating) and headaches. There were no reports of any adverse events in 83% of participants in the *Oroxylum indicum* group and 87% of participants in the placebo group. There were no statistically significant between-group differences in changes in systolic (*p* = 0.598) or diastolic (*p* = 0.700) blood pressure over time. However, there was a trend for a greater weight loss in the *Oroxylum indicum* group compared to the placebo group (*p* = 0.058), with a weight loss of 1.1 kg and 0.13 kg, respectively, from baseline to week 12.

**Table 7:** Frequency of self-reported adverse events.

	Placebo	*Oroxylum indicum*
Headache		2
Loose stools	2	1
Nausea		2
Bloating	1	2
Skin rash	1	1
Increased thirst	2	
Worsened sleep		1
Shortness of breath		1
Total number of	6	10

## Discussion

In this 12-week randomized, double-blind, placebo-controlled trial, supplementation with 500 mg twice daily of an *Oroxylum indicum* extract (Sabroxy^®^) in older adults with self-reported memory complaints was associated with greater improvements in episodic memory and several computer-based cognitive tasks compared to the placebo. Episodic memory was assessed by performance on several cognitive tasks including immediate word recall, delayed word recall, location learning recall, word recognition, picture recognition, numeric working memory, and the MoCA memory index score. In relation to changes in performance on individual tasks, there were greater improvements in immediate word recall and numeric working memory, and a faster rate of learning on the location learning task in participants supplemented with *Oroxylum indicum* compared to the placebo. However, there were no other significant differences in performance on the other assessed computer-based cognitive tests or the MoCA total score. Moreover, there were no significant between-group differences in changes in the CFQ, a self-report assessment of cognitive and memory skills, and the CASP-19, a quality-of-life measure for older adults. BDNF concentrations increased significantly in both groups, with no statistically-significant between-group differences. *Oroxylum indicum* was well tolerated except for a tendency for more mild digestive complaints and headaches. There was also a trend suggesting minor weight loss, although this finding requires verification in future clinical trials. In an *in vitro* trial, oroxylin-A was shown to inhibit adipogenesis (Singh and Kakkar, 2014).

Episodic memory refers to a neurocognitive system that makes possible the conscious recollection of events as they were previously experienced. It is a type of memory that allows us to re-experience personal events from the past. It involves the ability to learn, store, and retrieve information about personal experiences that occur in daily life and is believed to be influenced by neural components in the cortex near the hippocampus (perirhinal cortex, the entorhinal cortex, and the parahippocampal cortex), cortical and subcortical structures, and circuits within the medial temporal lobe and hippocampus (Camina and Guell, 2017). Individuals diagnosed with mild cognitive impairment and Alzheimer’s disease typically have severe deficits in episodic memory and dysfunction in the hippocampus (Nordahl et al., 2005). There is research to suggest that impairment of episodic memory may precede dementia by as much as 10 years (Dannhauser et al., 2008).

*Oroxylum indicum* may positively influence episodic memory *via*several mechanisms. As measurements of plasma BDNF concentrations in this study revealed there were no significant between-group differences, other mechanisms of action are speculated and require investigation in future trials. Concentrations of plasma F2-isoprostane are a reliable index of systemic oxidative stress (specifically lipid peroxidation) and are elevated in adults with dementia (Bradley-Whitman and Lovell, 2015). In a study on healthy older-age adults, higher F2-isoprostane concentrations were associated with significantly-worsened performance in episodic memory (Downey et al., 2018). In fact, the detrimental effects of oxidative stress on the cognitive decline have been regularly reported in the literature (Salim, 2017; Kandlur et al., 2020). *Oroxylum indicum* has strong antioxidant activity and its positive effects on episodic memory may be *via*its antioxidant effects and ability to inhibit lipid peroxidation (Siriwatanametanon et al., 2010). An inverse relationship between inflammation and cognitive performance has been demonstrated in several studies (Sartori et al., 2012). Although the results are inconsistent, higher inflammation is associated with worsened episodic memory (Simen et al., 2011; Tampubolon, 2016). The anti-inflammatory effects associated with *Oroxylum indicum* and its components, therefore, present another mechanism associated with its cognitive-enhancing effects (Kalaivani and Mathew, 2009; Tran et al., 2015; Lalrinzuali et al., 2016). The importance of neurotransmitters in memory has been demonstrated in several trials. For example, dopamine and GABA are neurotransmitters believed to be associated with attention, concentration, learning, memory, and mood (Kulisevsky, 2000; Mohler, 2009). In an acute study on older-age adults, administration of the dopamine precursor levodopa led to a dose-dependent benefit on episodic memory (Tampubolon, 2016). There is also accumulating evidence from human research indicating that midbrain dopamine regions modulate episodic memory, which occurs *via*interactions with the hippocampus (Shohamy and Adcock, 2010). Using H-magnetic resonance spectroscopy, a relationship between GABA concentrations in the medial prefrontal cortex and episodic memory has been observed (Thielen et al., 2019). GABA-A receptors are believed to play a key role in cognitive processes as the benzodiazepine receptor complex controls acetylcholine release in the hippocampus, and it has been demonstrated that GABA-A receptor antagonists increase acetylcholine release in the hippocampus and basal forebrain (Moor et al., 1998; Collinson et al., 2002; Vazquez and Baghdoyan, 2003). *In vitro* studies conducted on oroxylin-A (a chemical compound in *Oroxylum indicum*) have demonstrated that it can inhibit dopamine re-uptake (Dela Pena et al., 2013; Yoon et al., 2013), and has an antagonistic effect on the GABA-A receptor (Kim et al., 2008).

The location learning task is a measure of visuospatial learning and recall. Performance on this task is impaired in older adults, adults with dementia, and stroke patients (Bucks and Willison, 1997; Kessels et al., 2006). In this study, *Oroxylum indicum* was associated with a faster rate of learning compared to the placebo. How *Oroxylum indicum* influences spatial learning requires further investigation; however, several potential mechanisms are speculated. Because deficits in spatial memory can occur after hippocampal damage (Kessels et al., 2004) or lesions in the parietal cortex (Kessels et al., 2002; Schott et al., 2019), *Oroxylum indicum* may affect neural activity in these regions. Moreover, a relationship between visuospatial memory and cholinergic and dopaminergic neurotransmitter activity has been identified and, therefore, presents as another mechanism of action (Meador et al., 1993; Winkler et al., 1995). However, before definitive conclusions can be made on how *Oroxylum indicum* affects visuospatial learning, more research is required.

An interesting finding was the increase in plasma BDNF concentrations over time in both groups, with no statistically-significant between-group differences. Moreover, in this study, increases in BDNF were not associated with improvements in any cognitive outcome measure. Even though BDNF is believed to have an important role in synaptic plasticity and cognitive function, its measurement *via*human cognitive trials has revealed inconsistent findings (Lu et al., 2014; Miranda et al., 2019). However, low blood BDNF concentrations are more consistently identified in adults with Alzheimer’s disease, and less so in adults with mild cognitive impairment (Xie et al., 2020). The recruitment of adults with self-reported cognitive impairment in this study may, therefore, account for these non-significant findings. Lifestyle habits and genetic BDNF polymorphisms, which were not adequately controlled for in this study, may also account for the non-significant between-group findings. Approximately 50 percent of participants were taking a pharmaceutical medication and 30% were taking a nutraceutical which may have also impacted changes in BDNF over time. Moreover, even though research suggests there is an association between BDNF and cognitive function, there is limited evidence to demonstrate increasing plasma BDNF is associated with improvements in cognitive function. It is important to note that a possible explanation for the increase in BDNF in both groups may be due to recruitment timing. Participants had baseline assessments conducted in early spring and follow-up assessments in summer. This seasonal change may be associated with increased physical activity and sunlight exposure. Both physical activity (Szuhany et al., 2015) and sunlight exposure (Molendijk et al., 2012) can increase BDNF concentrations.

## Limitations and Directions For Future Research

Even though there were some positive results from this first human trial examining the cognitive enhancing effect of *Oroxylum indicum* on adults with self-reported cognitive complaints, further research is required to validate and extend these findings. In particular, it will be important to examine the efficacy and safety of *Oroxylum indicum* administered for longer periods, at different dosages, and on diverse populations including older adults with formally diagnosed mild cognitive impairment. It is important to note that the mean age of participants was 68 years which is a relatively young cohort. It is estimated that there is a prevalence of 8%, 10%, 15%, and, 25% of mild cognitive impairment in the age ranges of 65–69 years, 70–74 years, 75–79 years, and 80–84 years, respectively (Petersen et al., 2018). Since advanced age is associated with a greater risk of mild cognitive impairment and dementia, a greater emphasis on adults of more advanced age will be important in future trials to understand the effects of *Oroxylum indicum* on neurocognitive performance.

In this study, cognitive-enhancing benefits were observed after 3 months; however, it will be important to assess the safety and efficacy of at least 6 months of intake in older-age adults. Longer periods between assessments will also reduce the confounding influence of learning effects from multiple computer-based administrations. It is important to note that approximately 50 percent of participants were taking a pharmaceutical medication and 30% were taking a nutraceutical. While these factors were controlled for in the statistical analyses, it will be useful in future trials to examine the effects of *Oroxylum indicum* on medicated and unmedicated participants as cognitive performance and biological mechanisms such as acetylcholinergic activity can be modified by many medications and nutraceuticals (Colucci et al., 2012; Ruxton et al., 2015). This trial was designed to examine the chronic effects of *Oroxylum indicum* intake, so its acute cognitive effects could not be determined. As the 12-week cognitive assessment was conducted in the morning, approximately 12–16 h after the last intake of *Oroxylum indicum* (participants were instructed to not take the capsule on the morning of the assessment), the effects observed were unlikely to result from the acute administration of *Oroxylum indicum*. A computer-based cognitive test and a self-report measure were used to assess changes in cognitive performance. In future trials, it will be important to include ecologically validated outcome measures, particularly those associated with everyday performance and independent living. Moreover, additional outcome measures completed by clinicians and other informants will help validate the findings from this study. Further objective assessments including magnetic resonance imaging, single-photon emission computed tomography, and blood collections assessing markers of inflammation, oxidative stress, and other relevant hormonal activity will also be useful. Even though there were no between-group differences in changes in BDNF concentrations, the effects of a longer intake of *Oroxylum indicum*, and greater control on factors that may influence BDNF, may be worthwhile.

In summary, the results of this first human trial on the cognitive-enhancing effects of *Oroxylum indicum* suggest it is a promising herbal candidate for the improvement of cognitive function in older adults with self-reported cognitive complaints. The 12-week intake of 500 mg twice daily of an *Oroxylum indicum* extract (Sabroxy^®^) was associated with greater improvements in episodic memory and a faster rate of learning on the location learning task (a measure of visuospatial learning and recall). *Oroxylum indicum* was well-tolerated with no significant adverse effects although there was a tendency for increased reports of mild digestive complaints and headaches. Future investigations will be important to validate these findings and examine the safety and efficacy of *Oroxylum indicum* on different populations, adults of more advanced age, for longer treatment periods, at varying doses, and in populations where pharmaceutical medication use is more vigorously controlled. As *Oroxylum indicum* did not increase BDNF concentrations more than the placebo, further research into its potential mechanisms of action will be important.

## Data Availability Statement

The raw data supporting the conclusions of this article will be made available by the authors, without undue reservation.

## Ethics Statement

The studies involving human participants were reviewed and approved by National Institute of Integrative Medicine. The patients/participants provided their written informed consent to participate in this study.

## Author Contributions

AL and PD designed the research and analyzed the data. AL and SS conducted the research. AL, PD, SS, and MM wrote the article. AL has primary responsibility for the final content. All authors read and approved the final manuscript. All authors contributed to the article and approved the submitted version.

## Conflict of Interest

This study received funding from Sabinsa Corporation. The funder had the following involvement with the study writing of the article and approval of study design. AL is the managing director of Clinical Research Australia, a contract research organization that has received research funding from nutraceutical companies. AL has also received presentation honoraria from nutraceutical companies. SS is an employee of Clinical Research Australia and declares no other conflicts of interest. MM is the founder and chairman of the Sami-Sabinsa Group (financial sponsor of this trial). The remaining author declares that the research was conducted in the absence of any commercial or financial relationships that could be construed as a potential conflict of interest.

## Publisher’s Note

All claims expressed in this article are solely those of the authors and do not necessarily represent those of their affiliated organizations, or those of the publisher, the editors and the reviewers. Any product that may be evaluated in this article, or claim that may be made by its manufacturer, is not guaranteed or endorsed by the publisher.

## Footnotes

^1^ http://www.randomization.com
